# Systematic Review of Total Hip Arthroplasty Outcomes in Cerebral Palsy Patients and a Comparative Analysis with Rheumatoid Arthritis

**DOI:** 10.1155/2023/8696116

**Published:** 2023-12-30

**Authors:** Christos Costa, Foteini Moniati, Michalis Chatzimatthaiou, Christos Papaioannou, Sapfo Athanasakopoulou, Marios Chatzimatthaiou

**Affiliations:** ^1^Imperial Collage London, London, UK; ^2^Barts and the London School of Medicine and Dentistry—Queen Mary University of London, London, UK; ^3^Barts Health NHS Trust, London, UK; ^4^Chelsea and Westminster NHS Foundation Trust, London, UK; ^5^North Middlesex NHS Trust, London, UK

## Abstract

**Background:**

Total hip arthroplasty (THA) is considered a successful treatment option for patients with degenerative hip arthritis. However, in the setting of neuromuscular diseases, patients with cerebral palsy (CP) are considered high-risk due to instability, contractures, and altered muscle tone. The purpose of this systematic review is to analyse the data in the setting of THA in CP patients including indications, types of implants, revision rates, and patient-reported outcomes and compare these with those of a cohort requiring THA due to degenerative arthritis unrelated to neuromuscular disorders.

**Methods:**

PubMed, Embase, and Cochrane Library were searched from inception until June 10, 2023, to identify the relevant studies for THA on CP patients. The methodological quality of the studies was evaluated using the Newcastle–Ottawa Quality Assessment Scale (NOS).

**Results:**

The initial search generated 190 studies out of which 21 met the inclusion criteria. The most frequently reported indication was painful hip dislocation or subluxation due to failure of prior nonoperative treatment. The most frequently reported complication was dislocation affecting overall 7.5% (0–28%) of all patients, while other complications included periprosthetic fractures and heterotrophic ossification. The survival rates of primary THAs ranged from 85% to 100% at 5 years and from 73% to 86% at 10 years. Patients with CP who undergo total hip arthroplasty experience a greater overall rate of complications compared to patients with rheumatoid arthritis (RA) that undergo the same procedure.

**Conclusion:**

The current literature suggests that THA is a beneficial procedure for patients with CP through pain reduction and functional improvement. However, the increased rates of potential complications compared to the general population require careful consideration. We suggest that further investigations on the most appropriate time of procedure, implant type, and procedure are needed.

## 1. Background

First proposed by Dr. Little in 1853, cerebral palsy (CP) is a permanent yet common disorder, affecting balance, posture, and movement, with a prevalence of 2 in 1,000 live births [1]. Patients with CP may develop articular deformities such as increased femoral anteversion, hip dysplasia, and eventual dislocation due to muscle imbalance around the hips [2, 3]. Furthermore, previous studies have established that fifty to seventy-five percent of patients with CP experience secondary degenerative hip arthritis, associated gait disturbances, and difficulties with perianal hygiene [3]. In the younger population, the current literature suggests that nine out of ten children classified as Gross Motor Function Classification System (GMFCS) level V will experience hip dislocations [4].

Excisional arthroplasty has traditionally served as the gold standard treatment for reducing pain and functional limitations and improving gait [5]. Extensive research in the academic community has shown that ninety percent of patients with severe hip dislocation experience pain relief through this technique [3, 6]. However, in cases of spastic paralysis, this method has led to complications in twenty-four percent of cases [3]. Alternative salvage procedures, such as hip fusion, have yielded worse functional outcomes and higher complication rates [6, 7].

Alternative procedures for hip preservation and reconstruction, such as joint replacement, have also been proposed [8, 9]. Specifically, prior research supports the notion that total hip arthroplasty (THA) in patients with CP can improve gait, motion, and activity and provide pain relief [10]. Nevertheless, THA in this patient group presents unique surgical challenges, including issues with paresis, contracture, and muscular imbalance [11]. Moreover, concerns about aseptic loosening and joint dislocation have historically deterred orthopaedic surgeons from offering this procedure to patients with CP [5]. Despite the inherent risks, THA could prove beneficial for wheelchair-bound patients who can stand or transfer and for ambulatory patients.

However, there are limited and inconsistent data regarding perioperative protocols and patient outcomes, while complication rates remain high for neuromuscular patients undergoing THA. Within this context, this systematic review aims to critically analyse the data concerning THA in CP patients. This analysis will include indications, types of implants, revision rates, and patient-reported outcomes. Furthermore, it aims to compare these outcomes and related complications with those of a cohort requiring THA due to degenerative arthritis unrelated to neuromuscular disorders [12].

## 2. Methods

### 2.1. Literature Search Strategy

The Preferred Reporting Items for Systematic Reviews and Meta-Analyses (PRISMA) checklist and flow diagram were followed. The systematic search of the online databases PubMed, Embase, and Cochrane Library was conducted from inception until June 10, 2023, to identify the relevant studies for THA on CP patients. To make sure we included all the relevant studies, the appropriate MeSH search terms were used to devise a universal search question: (“total hip arthroplasty”/exp OR “total hip arthroplasty” OR ((“total”/exp OR total) AND (“hip”/exp OR hip) AND (“arthroplasty”/exp OR arthroplasty))) AND (“cerebral palsy”/exp OR “cerebral palsy” OR (cerebral AND (“palsy”/exp OR palsy))).

### 2.2. Inclusion and Exclusion Criteria

Our study employed specific inclusion criteria, with a primary focus on research pertaining to total hip arthroplasty (THA) in patients with cerebral palsy (CP). We narrowed our search to studies published in the English language and accessible as full-text articles in peer-reviewed journals. We excluded case series, reports, review articles, randomized controlled trials, and studies for which we did not have access to the complete paper.

### 2.3. Data Extraction and Critical Appraisal

The process of study selection was carried out independently by two of the authors at the outset. A consensus on the research question was reached, and MeSH terms were employed for the screening process. Initially, the authors screened the titles and pertinent abstracts, and when further evaluation was deemed necessary, the entire paper was comprehensively reviewed. In cases where disagreements arose concerning the inclusion of particular studies, a third independent author was consulted. This author implemented a voting system to determine whether the study in question should be incorporated or excluded from the analysis.

Two independent reviewers diligently collected and transcribed data from the eligible studies into an electronic screening form. The data extracted from each article encompassed various aspects, including authors' names, study title, publication year, study type, sample size, demographic characteristics, surgical indication, surgical details (such as the nature of the procedure, surgical approach, type of acetabular and femoral reconstruction, bearing surface, and additional procedures), as well as follow-up data (including the use of postoperative braces, follow-up duration, outcome measures, complications, revision surgeries, and implant survival). The eligible studies employed different tools for outcome assessment, and the data transcribed from these studies exhibited heterogeneity. Consequently, the results were predominantly qualitative in nature. As a result, conducting a meta-analysis with more advanced statistical analyses proved to be unfeasible.

## 3. Results

### 3.1. Description of Studies

**Figure 1:**
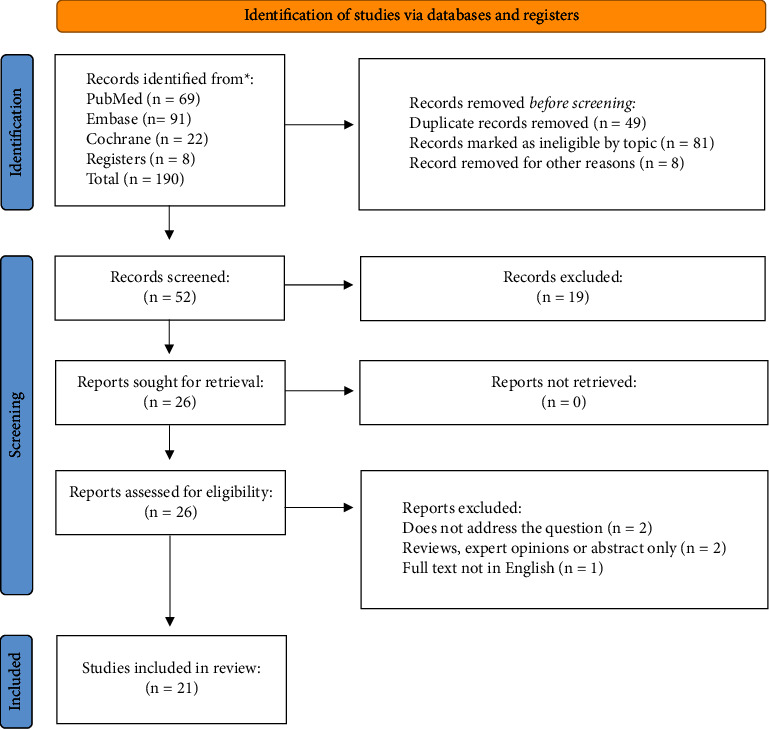
PRISMA flow diagram of the selected studies.

There were 190 studies collated in the initial database search. After the removal of duplicates, ineligible by topic records or those removed for other reasons, the number was subsequently reduced to 52. Following abstract screening, 26 reports were assessed for eligibility, leaving 21 studies included in the final review. The PRISMA flowchart with reasons for exclusion is illustrated in Figure 1.

### 3.2. Baseline Characteristics

This review included a total of 4,29,585 patients, while there was a total of 4886 THAs in CP patients. Also, the total number of hips replaced varied between studies from five to 2062.  Table 1 provides a summary of the characteristics of the selected studies. The majority of the studies were single-centred (86%), while 3 studies were conducted on multiple centres in the USA and England. Patient age was evaluated in 16 studies with the mean age ranging from 5 to 56.3 years of age. Sixteen studies reported on gender resulting in a 30.5 : 1 female-to-male ratio. Furthermore, controls were matched in three studies, while seventeen studies contained no controls. One study reported that there was no follow-up period, while the follow-up period reported ranged from 3 months to 10.5 years in the rest of seventeen studies.

Both the measure and assessment method of the outcome were diverse along the selected studies. The most commonly reported outcomes included postoperative complications, pain relief as well as revision rates. Furthermore, postoperative functional status was reported in 14 studies. Other outcomes included dislocation and reoperation rates, gait assessment, radiographic results, quality of life, and clinical status.

### 3.3. Methodological Quality Assessment

Two authors independently evaluated the quality of the studies included in this review. Fifteen of the studies were retrospective cohort studies and therefore were evaluated using the Newcastle–Ottawa Quality Assessment Scale (NOS) [24]. The abovementioned scale is designed to evaluate the quality of cohort studies or research via selection, comparability, and outcomes of the studies. More specifically, for the contents of selection (four numbered items) and outcome (three numbered items), each evaluated study was considered to be a maximum as one-star (^*∗*^) for every numbered item. As far as the contents of comparability are concerned, each study was considered to be a maximum of two-starts for every numbered item. The higher the total number of stars, the higher the quality of the study [25]. In the instance of unresolved disagreements, the reviewers judged and resolved them by consensus.  Table 2 shows the Newcastle–Ottawa Quality Assessment Scale results for the included studies.

### 3.4. Surgical Characteristics

The indication to perform THA was stated in 13 out of the 21 studies [8–10, 14–17, 19–23, 29]. The majority of the procedures were elective except from one emergency THA performed on a patient with femoral neck fracture [22]. The most frequently reported indication was painful hip dislocation or subluxation due to failure of prior nonoperative treatment. Other indications include treatment failure for prior femoral neck fractures and secondary arthritis.

Table 3 provides a summary of the studies that look at the surgical outcomes for patients with CP. Fourteen of those studies have included detailed analysis of the surgical procedure used. The most frequent one used was the posterolateral approach which was employed in six studies, including Buly et al. [31] and Raphael et al. [8] who have utilized both the transtrochanteric and posterolateral approach. Particularly, both Buly et al. [31] and Raphael et al. [8] have employed the transtrochanteric approach in 14 instances, whereas the posterolateral approach was used in 5 and 45 arthroplasties, respectively. Additionally, Silverio et al. [17], Morin et al. [10], Schroeder et al. [23], and Root et al. [32] applied the lateral approach, while Molenaers et al. [15], Abu-Rajab et al. [27], and Schorle et al. [28] adopted the standard anterior approach. Abousarma et al. [19] and Prosser et al. [9] opted for the posterior approach, and Gabos et al. [30] chose the anterolateral approach. Houdek et al. [16] employed both the anterolateral and posterior approach.

Information about the type of implant fixation utilized was reported by several studies. Particularly, for the fixation of the acetabular component, for studies have reported using uncemented cups only [15, 16, 20, 21], six studies reported the use of both cemented and uncemented cups [8, 10, 18, 23, 29, 30], while only two studies by Prosser et al. [9] and Buly et al. [31] have reported using cemented techniques exclusively.

Further reference was made by Molenaers et al. [15], which reported the use of dysplasia stems in 24 subjects due to distorted anatomy of the femur. Three studies have also employed the use of dual-mobility prosthesis. More specifically, Houdek et al. [16] employed dual-mobility acetabular constraints in 5 patients and lipped liner in 2. At the same time, Morin et al. [10] mentioned the use of 33 specialized dual-mobility uncemented acetabular components. Sanders et al. [22] reported the use of a dual-mobility cup (Avantage®, Biomet, Warsaw, IND) in all patients, consisting of a polyethylene liner articulating with both the femoral head and a fixed metal acetabular shell.

Furthermore, several studies have included information about additional surgical procedures that were required and postoperative interventions. These include adductor, hamstring tenotomies, and tendon lengthening. The study by Schroeder et al. has performed most soft tissue procedures with the majority being adductor tendon tenotomies and psoas or rectus tendon releases.

One significant aspect highlighted in the literature is the use of spica casts as a preventive measure against dislocation. Most study authors concurred on the standard indication for postoperative immobilization with an abduction brace or spica cast, particularly for patients who had experienced a dislocated hip prior to surgery. However, the effectiveness of spica casts in preventing dislocation remains a subject of debate. Schroeder et al. [23] found no correlation between THA instability and postoperative immobilization, despite the continued use of spica casts in patients with preoperative hip dislocation. Weber et al. [29] also utilized spica casts for patients with previous native dislocation. Additionally, Raphael et al. [8] and Buly et al. [31] introduced standardized postoperative casts following the occurrence of hip dislocations in their cases. It is noteworthy that hip dislocations were reported in all studies, irrespective of the prophylactic use of casts or braces.

### 3.5. Functional Outcomes

The functional assessment of patients was measured using the GMFCS [33]. Oxford Hip Score, Harris Hip Score, and EQ 5D Health Scale and Index included additional assessment methods. Pain levels were evaluated using self-developed questionnaires and the visual analogue scale.  Table 4 provides a summary of the functional outcomes of the selected studies. More specifically, positive pain relief outcomes were reported in 15 studies [8–10, 15–17, 19, 20, 23, 27–32]. Despite that the studies included varying patient populations, they universally demonstrated substantial improvements in pain levels postoperatively. Impressively, majority of the patients reported complete pain relief in 9 out of these studies [8–10, 16, 19, 23, 28, 30, 32]. Improved mobility and functional status postsurgery were highlighted in 11 studies [8–10, 16, 19, 20, 23, 28–31] where patients experienced enhanced independence in activities such as sitting, ambulation, and transferring. An improvement in the range of motion (ROM) and more specifically in hip flexion was noted in 3 of the studies [10, 16, 17], while Abousamra et al. [19] stated that there was no significant difference in the ROM.

### 3.6. Complications and Hip Survival Rates

Complications following total hip arthroplasty (THA) were prevalent in patients with cerebral palsy.  Table 5 provides a summary of the studies reporting complications. The most frequently documented complication was dislocation, affecting a total of 7.5% (ranging from 0% to 28%) of all patients. The second most common complication was periprosthetic fractures, observed in 5.6% of patients (with a range from 0% to 21%). Heterotrophic ossification was the third most common complication, occurring in 4.2% of patients (with a broad range of 0% to 37.8%). It is worth noting that heterotrophic ossification predominantly affected the paediatric and early adolescent population, potentially limiting its relevance to THA in adults with cerebral palsy.

Less frequently encountered complications encompassed acetabular/femoral loosening in 3.7% of patients (ranging from 0% to 15.4%) and surgical site infections in 2.1% of patients (with variability from 0% to 16.6%).

The need for revision surgery was emphasized in multiple studies, with an average of 8.8% of patients requiring further corrective surgical intervention. However, the rate of revision surgery exhibited significant variability across studies, ranging from 0% to as high as 19%.

With regard to the survival rates: of 13 (62%) studies that reported THA survival rates [8–10, 13, 15, 16, 18–20, 22, 23, 29, 31], the minority (3, 23%) reported the survival rates for at least two time points [8, 16, 18], while the majority (10, 77%) reported the survival rate at a single time point [9, 10, 13, 15, 19, 20, 22, 23, 29, 31].

**Figure 2:**
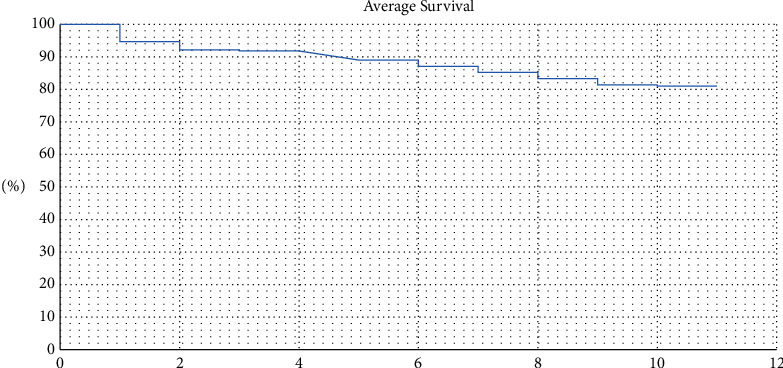
10-year survival of THA in patients with CP.

Survival rates were reported at different timeframes depending on the follow-up. The survival rates of primary THAs ranged from 85% to 100% at 5 years and from 73% to 86% at 10 years and were expectedly lower at 15 years (81%). The studies conducted after 2010 reported higher survival rates of primary THAs than those conducted from 1990 to 2000 and 2000 to 2010, reflecting on the modern implants and techniques used after 2010.  Figure 2 shows the forest plots of average survival rates.

### 3.7. Comparison of Complications in THA for Patients with CP and Rheumatoid Arthritis

**Figure 3:**
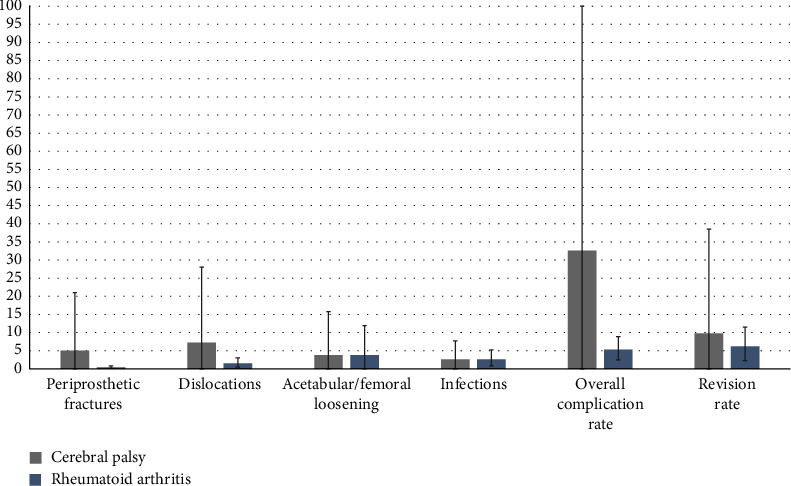
Illustration of the complications in THA for patients with CP vs. rheumatoid arthritis.

In our scientific investigation, we aimed to compare complication rates between patients with CP undergoing THA and patients with rheumatoid arthritis (RA) undergoing the same procedure. To achieve this, we conducted a comprehensive analysis of the data extracted from our systematic review and juxtaposed it with the findings of William's et al., who conducted a systematic review summarizing complication rates in RA patients. Our primary focus centered on four specific complications: periprosthetic fractures, dislocations, acetabular/femoral loosening, and infections. Additionally, we reported the overall complication rate and summarized the revision rate.  Figure 3 illustrates the comparison of the complications in THA for patients with CP and RA.

Our investigation revealed that patients with CP undergoing THA experience a higher overall complication rate when compared to their RA counterparts. This disparity is particularly evident in the increased incidence of periprosthetic fractures, dislocations, overall complications, and the necessity for revision procedures. Intriguingly, despite the fact that CP and RA patients undergoing THA exhibit similar average rates of infections and acetabular/femoral loosening, CP patients still present a higher incidence of these complications. This discrepancy is visually depicted by the wider error bars in Figure 3, which illustrate greater data variability and uncertainty surrounding the average values. In essence, these expanded error bars, observed across all complications in CP patients in comparison to RA patients, suggest both heightened data variability and an elevated likelihood of complications in CP patients [12].

It is essential to note that while the occurrence of infections in both patient groups is found to be at 2.6%, it is crucial to acknowledge the scope of the term “infections” [12]. In our study, we categorized surgical site infections and pneumonias under the umbrella of infections. Consequently, this categorization may not fully represent the complete spectrum of infections that manifest as complications in CP patients. This observation also raises the possibility that infection rates in CP patients may indeed surpass those in RA patients.

However, despite the overarching conclusion that CP patients generally experience a higher rate of complications than RA patients, a significant challenge arises from the considerable variability in the assessment duration for revision rates. Different studies incorporate diverse follow-up periods, ranging from a few months to several years [16]. This variation in follow-up durations presents a substantial challenge when attempting to make definitive conclusions about the differences in complications. It becomes difficult to ascertain whether these differences are primarily attributed to the patient's underlying condition or are influenced by the varying follow-up durations.

## 4. Discussion

Spastic hip dysplasia presents a significant challenge for individuals with CP, with previous research indicating that 75% of CP patients suffer from degenerative hip arthritis, reduced mobility, pain, and perineal care difficulties [34].

Our findings affirm the viability of total hip arthroplasty (THA) as a treatment option for CP patients, offering substantial benefits. However, it is crucial to approach each case individually to ensure the best possible outcome.

In this comprehensive review of 21 studies related to total hip arthroplasty (THA), several key findings emerged. THA indications were specified in 13 of these studies, with the majority of procedures being elective, except for one emergency THA performed in response to a femoral neck fracture. The most commonly cited indication for THA was the presence of painful hip dislocation or subluxation, often resulting from failed nonoperative treatments. Additional indications included treatment failure for prior femoral neck fractures and the development of secondary arthritis.

Regarding surgical approaches in THA, it is notable that the anterior approach and posterolateral approach were the most frequently employed methods, each reported in four studies. Following closely were the lateral and posterolateral approaches, each used in three studies, respectively. As for prosthesis types, the uncemented approach predominated in eight out of nine studies, followed by cemented prostheses in five of the nine selected studies. Two studies reported the use of hybrid prostheses, and one utilized reverse hybrid prosthesis.

An important aspect highlighted in the literature is the use of spica casts as a preventive measure against dislocation. While most authors agreed on standard postoperative immobilization with an abduction brace or spica cast, especially for patients with preoperative hip dislocation, the effectiveness of these measures remains a topic of debate. Some studies found no clear correlation between THA instability and postoperative immobilization, yet spica casts continued to be employed, particularly in cases with a history of hip dislocation. In summary, it is noteworthy that hip dislocations were reported in all studies, regardless of the use of prophylactic casts or braces. These findings offer valuable insights into the indications, surgical approaches, and postoperative practices in the realm of THA, shedding light on areas of ongoing clinical inquiry and potential improvement.

Concerning complication monitoring, there was considerable variation in follow-up protocols across the studies, ranging from no follow-up to different timeframes. The most frequently reported complication was hip dislocation, followed by aseptic loosening, resulting in higher revision rates among cerebral palsy (CP) patients. Notably, no association was found between revision rates and the type of implant used, including both cemented and uncemented acetabular components.

Given that instability/dislocation remains one of the most common complications of THA, some authors have suggested that constrained liners (CL) may be associated with higher loosening rates in the long term [35]. However, Ryu et al. have shown that both CL and dual-mobility (DM) liners effectively reduce the risk of hip dislocation in elderly patients with neuromuscular diseases, including CP [36]. Historically, traditional materials such as metal-on-polyethylene have been utilized in THA. The recent advancements in orthopaedic biomaterials however have led to the exploration of alternative options, including ceramic-on-ceramic and metal-on-metal bearings, which may offer improved wear resistance and longevity. Nevertheless, there is a lack of prior studies examining the association between the size of the femoral head, treatment efficacy, and long-term performance of the different materials in CP patients undergoing THA [37]. Therefore, future research should not only confirm the optimal treatment strategy for CP patients but also identify the most effective type of acetabular insert and material type for this specific group of patients.

Postoperative revision rates displayed a wide range, spanning from 0% to 19%, while implant survivorship at the 10-year mark varied from 73% to 86%. It is worth noting that these rates fall below the 10-year survival rate typically observed in primary THA procedures. Consequently, further research is imperative to ascertain the long-term survival rates of THA in CP patients, as well as to investigate other complications such as blood loss and associated laboratory changes, along with their associated risk factors. These investigations will provide valuable insights into the outcomes and potential improvements in THA procedures for individuals with CP.

Moreover, we conducted a comprehensive analysis to compare complication rates between cerebral palsy (CP) patients undergoing total hip arthroplasty (THA) and rheumatoid arthritis (RA) patients undergoing the same procedure. By synthesizing data from our systematic review and contrasting it with findings from RA studies, we focused on four critical complications: periprosthetic fractures, dislocations, acetabular/femoral loosening, and infections, alongside overall complication and revision rates. Our investigation revealed higher complication rates in CP patients undergoing THA, particularly in periprosthetic fractures, dislocations, overall complications, and the need for revisions. Despite similar infection and loosening rates, CP patients exhibited more complications reflecting greater data variability and a heightened likelihood of complications. However, the uniform 2.6% infection rate may not fully encompass all infections in CP patients. The variability in revision rate assessment durations poses challenges in drawing definitive conclusions. Further research is needed to explore these complexities thoroughly.

In accordance with the transition toward value-based healthcare, the success of the examined studies was evaluated through patient-reported outcome measures. However, it was noted that commonly assessed outcomes, including complications, pain relief, and revision rates, were appraised using a diverse range of scoring systems. Despite this variation, it can be deduced that the overall functional assessment scores for CP patients undergoing THA were generally favourable. This finding aligns with the results of Adams et al., who similarly reported positive outcomes from THA in CP patients, including pain relief and improved caregiving hygiene [38]. Nevertheless, it is crucial to emphasize the need for additional research to substantiate these conclusions, as no assessment of methodological quality was conducted.

Our systematic review also has limitations. Foremost among these limitations is the small sample size in the reviewed studies, with 12 out of 21 studies having fewer than 20 patients. This diminishes the likelihood of obtaining statistically significant results and conducting power calculations effectively. Additionally, there were variations in study design apparent, including differences in surgical approaches chosen among studies, study populations, and follow-up times. While the posterolateral approach was favoured by most orthopaedic surgeons, some studies employed an anterolateral approach [16, 30], while others employed the lateral approach [10, 17, 23, 32]. Furthermore, the follow-up time ranged from no follow-up time to 10 years of follow-up reported in the studies [8, 20, 29, 31]. Moreover, as previously explained, the mean age of the population group in each study varied significantly, ranging from 5 to 59 years old. Lastly, several studies recruited patients from single centres, limiting the geographical diversity of the data and potentially affecting the generalizability of the findings, essentially resulting in selection bias.

## 5. Conclusion

In summary, this systematic review suggests that total hip arthroplasty (THA) represents a valuable treatment choice for individuals with cerebral palsy (CP) who are dealing with articular deformities, hip dislocation, and degenerative arthritis. However, owing to the scarcity of data available for this high-risk procedure in CP patients, further research on this approach is crucial to enhance outcomes. Future studies should aim to ascertain the optimal implant fixation methods and types of acetabular inserts, while also evaluating the long-term survival rates of THA.

## Figures and Tables

**Table 1:** Summary of the characteristics of the selected studies.

Study, year	Type of study	Study population	Number of patients	Age (mean)	M : F ratio	Controls matched	Follow-up(years)
Moore et al. [13], 2021	Cohort study	Multicentred: 2010–2018, USA	864	56	N/A	Yes	0.25
Moon et al. [14], 2020	Retrospective cohort study	Multicentred, USA 2005–2014	2062	45	N/A	No	No follow-up
Molenaers et al. [15], 2017	Retrospective cohort study	Single centre	29	35	13 : 16	No controls	3.25
Houdek et al. [16], 2017	Retrospective cohort study	Single centre, USA 1990–2013	119 (CP: 41 vs. non-CP: 78)	49	29 : 13	Yes	2
Silverio et al. [17], 2016	Retrospective cohort study	Single centre	12	12	9 : 3	No controls	2
Morin et al. [10], 2016	Retrospective cohort study	Single centre: 2001–2014, France	33	19	N/A	No controls	5.3 years
King et al. [18], 2016	Retrospective cohort study	Multicentred, England 2003–2012	426 202 (389 with CP)	53	159 : 230	Yes	Not specified
Abousamra [19], 2016	Retrospective cohort study	Single centre	12	14	10 : 2	No controls	4
Yoon et al. [20], 2015	Retrospective cohort study	Single centre, South Korea 2005–2007	5	36	3 : 2	No controls	N/A
Alosh et al. [21], 2014	Retrospective cohort study	Single centre	27	49	19 : 8	No controls	2.5
Sanders et al. [22], 2013	Cohort study	Single centre, Netherlands 2008–2010	10	54	6 : 4	No controls	3.2
Prosser et al. [9], 2012	Cohort study	Single centre	19	37	10 : 9	No controls	8.0
Schoreder et al. [23], 2010	Cohort study	Single centre, Germany 1988–2006	13	42	5 : 8	No controls	10.5
Raphael et al. [8], 2010	Retrospective cohort study	Single centre, USA 1972–2006	56	31	36 : 23	No controls	9.7 years [2–25]
Gavrakaptenovic et al. [26], 2007	Retrospective cohort study	Single centre, Serbia	31	5	8 : 23	No controls	12 months
Abu-Rajab et al. [27], 2007	Retrospective cohort study	Single centre	15	16	5 : 10	No controls	N/A
Schorle et al. [28], 2006	Retrospective cohort	Single centre	16	48	11 : 5	No controls	N/A
Weber et al. [29], 1999	Retrospective cohort	Single centre, USA	16	49	N/A	No controls	9.7
Gabos et al. [30], 1999	Retrospective cohort study	Single centre	11	17	6 : 5	No controls	5
Buly et al. [31], 1993	Retrospective cohort study	Single centre	18 (19 THA)	30	17 : 13	No controls	10
Root et al. [32], 1986	Retrospective cohort study	Single centre	15 patients (15 THA)	31	7 : 8	No controls	6.75

**Table 2:** Quality and risk of bias assessment using the Newcastle–Ottawa Scale (NOS).

Study ID	Selection				Comparability	Outcome			Total (9^*∗*^)
	(a)	(b)	(c)	(d)	(e)	(f)	(g)	(h)	
1. Moore et al. [13], 2021	1	1	0	1	2	1	0	1	7
2. Moon et al. [14], 2020	1	1	1	1	0	1	0	1	6
3. Molenaers et al. [15], 2017	1	0	1	1	0	1	1	1	6
4. Houdek et al. [16], 2017	1	1	1	1	2	1	1	1	9
5. Silverio et al. [17], 2016	1	0	1	1	0	1	1	1	6
6. Morin et al. [10], 2016	1	0	1	1	0	1	1	1	6
7. King et al. [18], 2016	1	1	1	1	0	1	1	1	7
8. Abousamra et al. [19], 2016	1	0	1	1	0	1	1	1	6
9. Yoon et al. [20], 2015	1	0	0	1	0	1	1	1	5
10. Alosh et al. [21], 2014	1	0	1	1	0	0	1	1	5
11. Sanders et al. [22], 2013	1	0	1	1	0	1	1	1	6
12. Prosser et al. [9], 2012	1	0	1	1	0	0	1	1	5
13. Schoreder et al. [23], 2010	1	0	1	1	0	1	1	1	6
14. Raphael et al. [8], 2010	1	0	1	1	0	1	1	1	6
15. Gavrakaptenovic et al. [26], 2007	1	0	1	1	0	0	1	1	5
16. Abu-Rajab et al. [27], 2007	1	0	1	1	0	1	1	1	6
17. Schorle et al. [28], 2006	1	0	1	1	0	0	1	1	5
18. Weber et al. [29], 1999	1	1	1	1	0	0	1	1	6
19. Gabos et al. [30], 1999	1	0	1	1	0	1	1	1	6
20. Buly et al. [31], 1993	1	0	1	1	1	1	1	1	7
21. Root et al. [32], 1986	1	0	1	0	0	1	1	1	5

(a) Representativeness of the exposed cohort (^*∗*^), (b) selection of the nonexposed cohort (^*∗*^), (c) ascertainment of exposure (^*∗*^), (d) demonstration that the outcome of interest was not present at the start of the study (^*∗*^), (e) comparability of cohorts (^*∗∗*^), (f) assessment of outcome (^*∗*^), (g) follow-up was long enough for outcomes to occur (^*∗*^), and (h) adequacy of follow-up (^*∗*^).

**Table 3:** Summary of the studies that look at surgical outcomes for patients with cerebral palsy.

Studies	Surgical approach	Cemented vs. uncemented	Additional procedures including soft tissue release	Head size	Bearing surface	Specialized implants (dual mobility)
Molenaers et al. [15], 2017	Anterior approach	100% uncemented	N/A	N/A		N/A

Houdek et al. [16], 2017	Anterolateral approach: 40% posterior: 60%	100% uncemented	9 tendon release: adductor [7] and psoas [2]	32 mm (22–40)		Acetabular constraint (dual mobility [5] or lipped liner [2])

Silverio et al. [17], 2016	Lateral approach	N/A	N/A	N/A		N/A

Morin et al. [10], 2016	Lateral approach	Cemented: 93% uncemented: 7%	Trochanteric osteotomy and neck section	26 mm	Meta-polyethylene	Dual mobility prosthesis with uncemented acetabular component [30] and cemented femur

King et al. [18], 2016	Not specified	Cemented: 18%, uncemented: 42%, hybrid: 22% reverse hybrid: 16%	N/A	N/A	MoP 36.5% MoM 39% Cop 13.6% CoC 21.4% CoM 0.5%	N/A

Abousamra et al. [19], 2016	Posterior approach	N/A	Combined femoral and acetabular bony reconstruction with femoral osteotomy fixation	N/A	N/A	N/A

Yoon et al. [20], 2015	Posterolateral approach	Uncemented: 100%	Release of flexion/adduction contracture of the hip, and tight repair of soft tissue	N/A	CoP	N/A

Alosh et al. [21], 2014	Posterolateral approach	Uncemented 100%	Hip flexor lengthening and hip adductor release	32 mm (range 22–32)	N/A	N/A

Sanders et al. [22], 2013	Posterolateral approach	N/A	Additional adductor tenotomy in 1 patient	N/A	N/A	Dual mobility including advantage, Biomet, Warsaw, IND

Prosser et al. [9], 2012	Posterior approach	Cemented: 100%:	Resurfacing and osteotomy procedure	N/A	N/A	N/A

Shroeder et al. [23], 2010	Lateral approach	Uncemented: 73%, cemented: 27%	(i) Adductor tenotomy	N/A	N/A	N/A
			(ii) Lengthening of adductor tendons			
			(iii) Psoas and rectus tendon release			

Raphael et al. [8], 2010	Posterolateral	Cemented: 20%, uncemented: 3% hybrid: 77%	Flexor or adductor tendon releases	22 or 32 mm	N/A	N/A

Gavrakaptenovic et al. [26], 2007	N/A	N/A	(i) Open reduction of the hip [10]	N/A	N/A	N/A
			(ii) Acetabuloplasty [19]			
			(iii) Shortening and osteotomy [13]			

Abu-Rajab et al. [27], 2007	Anterior approach	N/A	N/A	N/A	N/A	N/A

Schorle et al. [28], 2006	Anterior approach	N/A	(i) Detachment of the contracted pars	N/A		N/A
			(ii) Rectus femoris lengthening implantation of ARR support ring			

Weber et al. [29], 1999	N/A	Cemented: 87.5% uncemented 12.5%	2 tenotomies	N/A	N/A	N/A

Gabos et al. [30], 1999	N/A	Cemented and uncemented	Hamstring and adductor releases	N/A	N/A	N/A

Buly et al. [31], 1993	Transtrochanteric approach: 74%	Cemented: 100%	Flexor or adductor release	N/A	N/A	N/A
	Posterolateral approach: 26%					

Root et al. [32], 1986	Posterolateral approach: 13%	N/A	Soft tissue release	N/A	N/A	N/A
	Lateral approach: 87%					

**Table 4:** Summary of the functional outcomes of the selected studies.

Study, year	Number of patients	Functional outcomes:
Molenaers et al. [15], 2017	29	(i) Mean postoperative Harris Hip Score for 17 patients: 79 (range 56–97)
		(ii) Excellent outcomes observed in 13 patients
		(iii) Good outcomes achieved in 4 patients
		(iv) A total of 20 patients reported being good and very satisfied with the results
		(v) 5 patients expressed satisfaction with some limited pain

Houdek et al. [16], 2017	119 (CP: 41 vs. non-CP: 78)	(i) Preoperative pain: all 41 patients experienced moderate to severe pain, while postoperatively, none reported pain
		(ii) Hip flexion contracture of ≥ 15°: 9 patients had it before surgery, but none had it postop
		(iii) Ambulation status preop: 10% were independent, 70% used aids, and 20% did not use aids. Postoperatively, 54% were independent, 46% used aids, and none required aids
		(iv) Harris Hip Score (0–100): preop score was 36, while postop score improved to 78

Silverio et al. [17], 2016	12	(i) Overall outcomes: 7 hips rated as excellent/good, while 9 were rated as fair/poor
		(ii) Pain outcomes: 9 hips achieved excellent/good results, and 7 showed fair/good outcomes. The average pain reduction was 8.2/10 following PFIA
		(iii) Range of motion (ROM) outcomes: 9 hips had excellent/good ROM, and 7 hips had fair/poor ROM

Morin et al. [10], 2016	33	(i) Function: independent sitting improved from 5 patients (15%) preop to 6 (18%) postop
		(ii) Pain: permanent pain reduced from 16 patients before THR (40%) to none after THR. Pain while sitting decreased from 20 patients preop (50%) to 1 patient postop (2.5%), and pain during transfer decreased from 28 patients preop (70%) to none postop
		(iii) Range of motion: flexion exceeding 80 degrees increased from 19 patients before THR (47.5%) to 34 patients after THR (85%)

King et al. [18], 2016	426 202 (389 with CP)	(i) Oxford Hip Score (0–48):
		(a) CP group: 47 pairs, with 12 at preop and 34 at 6-month follow-up
		(b) Control group: 92,073 pairs, with 18 at preop and 41 at 6 months
		(ii) EQ-5D health scale (0–100):
		(a) CP group: 43 pairs, with a score of 60 preop and 70 at 6 months
		(b) Control group: 80,341 pairs, with a score of 70 preop and 80 at 6 months

Abousamra et al. [19], 2016	12	(i) Pain: preop pain rated at 9, postop pain reduced to 0
		(ii) Range of motion: no statistically significant changes observed
		(iii) Gait: no statistically significant changes detected
		(iv) Radiographic changes: femoral head deformity improved in 4 hips and remained the same in 8. In contrast, acetabular deformity improved in 11 hips and remained the same in just 1 hip
		(v) Top of form

Yoon et al. [20], 2015	5	(i) Mean leg length discrepancy improved from 4.3 cm (range, 1.2 to 8 cm)
		(ii) Pain: 3 patients experienced complete pain relief, while 2 patients saw a reduction in their preoperative pain
		(iii) Function: 3 patients were able to return to their GMFCS level prior to the onset of hip pain, and 2 patients regained their preoperative GMFCS levels

Prosser et al. [9], 2012	19	(i) 16 out of 18 hips (89%) were painfree after surgery
		(ii) GMFCS grade improved in six patients (32%) at the last follow-up
		(iii) Perineal access was enhanced in 11 out of 13 (85%) quadriplegic patients

Schoreder et al. [23], 2010	13	(i) Pain levels assessed using a numerical rating scale (NAS):
		(ii) No pain: 10 patients, grade 2 pain: 1 patient, grade 4 pain: 1 patient, grade 10 pain: 1 patient
		(iii) Mean pain decreased significantly from 8.4 preoperative to 1.1 postoperative (*p*=0.002)
		(iv) Walking outcomes:
		(a) 5 patients no longer required walking aids after the operation
		(b) 2 patients who initially walked without walking aids needed walking sticks after the operation

Raphael et al. [8], 2010	56	(i) Pain outcomes:
		(a) 81% (48 out of 56 patients) experienced complete pain relief
		(b) All patients achieved a reduction in preoperative pain
		(c) Mean pain score decreased from 8/10 to 0.7 postoperatively
		(ii) Function outcomes:
		(a) 88% (52 out of 56 patients) returned to their original GMFCS (gross motor function classification system) level of function before the onset of hip pain

Abu-Rajab et al. [27], 2007	15	(i) Pain:
		(a) Preop: 20 out of 21 patients (95%) reported pain
		(b) Postop: 9 out of 21 patients still experienced pain
		(ii) Seating difficulties:
		(a) Preop: 12 out of 21 patients (57%) had seating difficulties
		(b) Postop: only 1 out of 21 patients had seating difficulties after the operation

Schorle et al. [28], 2006	16	(i) 13 patients were pain-free; 3 patients had minor residual symptoms; independent mobility was improved

Weber et al. [29], 1999	16	(i) 79% of the patients reported improvement in function
		(ii) 87% reported good/excellent pain relief

Gabos et al. [30], 1999	11	(i) Pain: complete pain relief in 10 patients out of 11
		(ii) Sitting tolerance: improved in 11/11 patients, 5 patients achieved unlimited sitting tolerance

Buly et al. [31], 1993	18 (19 THA)	(i) 17 of 18 patients (94%) had pain relief and improved function after THA

Root et al. [32], 1986	15 patients (15 THA)	(i) 10/15 patients were pain-free

**Table 5:** Summary of studies reporting complications.

	Patients (no. of THA)	PPF	Dislocation (*n*, %)	AFL	SSI	Ossification	Other	RR	HS (CP/control) (years)
Moore et al. [13], 2021	864	8/0.9%	2.7%	0	14/1.6%	0	0	50/5.8%	5 y: 94.2%/95.2%
Moon et al. [14], 2020	2062	N/A	N/A	N/A	6/0.3%	N/A	Anaemia (30.2%)	N/A	N/A
Molenaers et al. [15], 2017	29/37 THA	1 (2.7%)	5, 13.5%	1 (2.7%)	1 (2.7%)	1 (2.7%)		4 (10.8%)	At 3 years: 89.2%
Houdek et al. [16], 2017	41	0	3, 7.3%	2 (4.8%)	1 (2.4%)	0	DVT: 1 (2.4%)	5/39 (12.8%)	2 y: 92%, 5 y 88%, 10 y 81%
Silverio et al. [17], 2016	12/16 THA	1 (6.3%)	0	0	0	6 (37.8%)	Anaemia (3.18.9%)	3/16 (19%)	N/A
Morin et al. [10], 2016	33/40 THA	3 (7.5%)	1, 2.5%	2 (5%)	2 (5%)	0	RDS (1.2.5%)	6 (15%)	5 y 85%
King et al. [18], 2016	389	N/A	N/A	N/A	N/A	N/A	N/A	N/A	−1 y 2.6%, 3 y 4.7%, 5 y 6.4%
Abousamra et al. [19], 2016	12	0	0	0	0	0	LLD [7]	0	90% at 3 years
Yoon et al. [20], 2015	5	1 (20%)	1, 20%	0	0	0	0	0	0 revisions
Alosh et al. [21], 2014	27/30 THA	1 (8.3%)	0	0	2 (16.6%)	0	Sacral ulcer (1, 8.3%)	0	
Sanders et al. [22], 2013	10	1 (10%)	0	0	0	0	0	1 (10%)	3 y 90%
Prosser et al. [9], 2012	19	1 (5%)	2, 10%	3 (15%)	0	0	RF (2.10%)	3 (15%)	8 y 85%
Schoreder et al. [23], 2010	13/15 THA	0	1, 7.7%	2 (15.4%)	1 (7.7%)	0	0	4 (27%)	10 y 73%
Raphael et al. [8], 2010	56/59 THA	1 (1.7%)	5, 8.5%	2 (3.3%)	1 (1.7%)	0		9 (15.3%)	2 y 95%, 10 y 85%
Gavrakaptenovic et al. [26], 2007	31/45 THA	2 (4.4%)	3, 6.6%	0	2 (4.4%)	0	0	4 (8.8%)	N/A follow-up info
Abu-Rajab et al. [27], 2007	15/21 THA	0	1, 4.7%	0	0	0	Anaemia [2], N&V [4]	1 (4.7%)	N/A follow-up
Schorle et al. [28], 2006	16	1 (6.3%)	1, 6.3%	0	0	0	N/A	0	N/A follow-up
Weber et al. [29], 1999	16	2 (12.5%)	0	1 (6.3%)	0	0	0	3 (18.3%)	82% at 10 years
Gabos et al. [30], 1999	11/14 THA	3 (21%)	4, 28%	2 (14%)	0	5 (35%)	0	0	N/A follow-up
Buly et al. [31], 1993	18/19 THA	0	2, 10.5%	0	0	0	0	2 (10.5%)	86% at 10 years
Root et al. [32], 1986	15	0	2, 13.3%	1 (6.7%)	0	0	Bursitis (3.20%)	1 (6.7%)	N/A

RR: revision rate, SSI: surgical site of infection, PPF: periprosthetic fractures AFL: acetabular femoral loosening, HS: hip survivorship, LLD: leg length discrepancy, and RF: respiratory failure.

## Data Availability

The data used to support the findings of this study are available from the corresponding author upon reasonable request.
